# A RFLP 1-4-3 L1C Variant of PRRSV-2 Isolated in Sichuan Province, China: Genetic Characterization and Pathogenicity

**DOI:** 10.1155/tbed/6817783

**Published:** 2024-12-12

**Authors:** Li-Shuang Deng, Zhi-Jie Jian, Yuan-Meng Wang, Bing-Zhou Huang, Tong Xu, Feng-Qin Li, Si-Yuan Lai, Yan-Ru Ai, Jian-Bo Huang, Zhi-Wen Xu, Ling Zhu

**Affiliations:** ^1^College of Veterinary Medicine, Sichuan Agricultural University, Chengdu, China; ^2^Sichuan Key Laboratory of Animal Epidemic Disease and Human Health, Sichuan Agricultural University, Chengdu, China

**Keywords:** cytokine, L1C variant, pathogenicity, phylogenetic analysis, PRRSV, recombination analysis

## Abstract

Porcine reproductive and respiratory syndrome virus (PRRSV), known for causing reproductive disorders in sows and respiratory issues in piglets, poses a significant threat to the global swine industry. Since its initial report in 2013, the L1C (lineage 1.8/NADC30-like) PRRSV has drawn significant attention in China due to its high recombination potential and diverse pathogenicity. This study focuses on a naturally occurring recombinant L1C variant, SCABTC-202302, characterized by an restriction fragment length polymorphism (RFLP) pattern of 1-4-3. We investigate the strain's genetic evolution, recombination, pathogenicity, and immune and antibody responses. Phylogenetic analysis of the ORF5 (open reading frame) gene classified the SCABTC-202302 strain as lineage 8.7, while whole-genome analysis categorized it as L1C. Notably, a discontinuous deletion of 131 amino acids (AAs) was observed in the NSP2 gene, along with specific AA mutations in ORF5. Recombination analysis revealed the NADC30 strain as the primary parent, with contributions from the JXA1 strain in the ORF2-ORF7 region. The strain caused lung and lymph node damage, sustained high-level viremia, and elevated inflammatory factors in infected piglets. Our study provides valuable insights into the genetic characteristics, pathogenicity, and immunological profile of L1C strains, contributing to the development of vaccines and control measures for PRRSV.

## 1. Introduction

Porcine reproductive and respiratory syndrome (PRRS) is a globally prevalent infectious disease in the swine industry, carrying substantial economic implications [1, 2]. The causative agent, PRRS virus (PRRSV), is an enveloped, positive-sense RNA virus categorized under the order Nidovirales and the family Arteriviridae [3]. It has a genome of ~15 kb, composed of a 5′-untranslated region (UTR), 10 overlapping open reading frames (ORFs), and a 3′-poly (A) tail [1, 4]. PRRSV is classified into two species: *Betaarterivirus suid* 1 (PRRSV-1) and *Betaarterivirus suid* 2 (PRRSV-2), represented by the LV and VR2332 strains, respectively [5–7]. Since its first identification in China, PRRSV-2 has predominated in our country [8].

PRRSV exhibits extensive genetic diversity, particularly in the ORF5 gene, which is commonly employed for genetic evolution studies [9]. Phylogenetic analysis of the ORF5 gene divides PRRSV-2 into nine lineages (lineages 1–9), with lineage 1 being globally distributed and genetically complex [10]. Recently, lineage 1 was further divided into eight sublineages (L1A–L1H) [11]. In China, the main prevalent sublineages are L1C (lineage 1.8/NADC30-like) and L1A (lineage 1.5/NADC34-like) [1]. Restriction fragment length polymorphism (RFLP) typing based on the cleavage patterns of *Mlu*I, *Hinc*II, and *Sac*II enzymes in the ORF5 in PRRSV-2 classification [12]. The NSP2 gene, also highly variable, exhibits strain-specific deletions or insertions of amino acids (AAs), such as the 131-AA deletion in L1C strains and the 100-AA deletion in L1A strains [13, 14].

One of the primary challenges in preventing and controlling PRRSV is its capacity for rapid recombination and mutation in response to environmental changes [9]. Since the first report of the PRRSV-2 strain in China in 1996, it has become widespread and continuously evolved in the country. In 2006, lineage 8.7 (HP-PRRSV-like) strains caused a large-scale outbreak, affecting over two million pigs in China [15]. L1C strains have spread widely since 2013 [16], and after 2017, L1A strains rapidly circulated nationwide [17]. The concurrent circulation of multiple PRRSV lineages in Chinese pig herds has accelerated viral recombination and mutation, resulting in various recombination patterns and new recombinant PRRSV-2 strains. These recombination events primarily involve the L1C, L1A, lineage 8.7, and lineage 5.1 (VR2332-like) strains [18]. Among these lineages, L1C strains exhibit higher recombination potential and greater pathogenic diversity, likely due to specific recombination regions [19, 20]. PRRSV strains are also known for their high mutation rates, particularly in the ORF5 and NSP2 genes, which are commonly monitored for PRRSV mutations [10, 14]. ORF5 gene encodes the structural protein GP5, which contains neutralizing antibody epitopes. Mutations in ORF5 may alter the antigenic properties and virulence of the strains [19].

Cytokines such as interferon-alpha (IFN-*α*), tumor necrosis factor-alpha (TNF-*α*), and interleukin (IL)-1 play crucial roles in inducing host responses, including fever, inflammation, histological changes, and potentially severe outcomes like shock or death [21]. PRRSV manipulates these immune responses and induces cytokine production in the lungs and blood [22, 23].

This study identifies a novel recombinant L1C strain of PRRSV-2, SCABTC-202302, isolated from a pig farm in Sichuan, China. A comprehensive analysis of its whole-genome characteristics, recombination events, pathogenicity, and immune and antibody responses was conducted. These findings enhance our understanding of the genetic variations and pathogenic mechanisms of L1C strains, contributing to the development of effective PRRSV prevention and control strategies.

## 2. Materials and Methods

### 2.1. Sample Collection and Detection

During May to June 2022, a pig farm in Sichuan Province, China, experienced an epidemic characterized by miscarriages in sows and high mortality among piglets (*Supplementary Figure 1*). The miscarriage rate in sows was 16%, and the mortality rate in piglets reached 7%. Clinical samples, including serum, lungs, and lymph nodes, were collected and homogenized in phosphate-buffered saline (PBS). Subsequently, total RNA was extracted from the supernatant and converted into cDNA using Trizol and the PrimeScript RT Reagent Kit following the manufacturer's instructions (Takara, Dalian, China). The samples were then analyzed using the PRRSV GP7-specific real-time quantitative PCR (RT-qPCR) assay, as previously reported by our laboratory [18].

### 2.2. Viral Isolation and Identification

PRRSV-positive samples were selected for subsequent viral isolation. The supernatant from these samples was filtered using a 0.22 μm filter and then inoculated into Marc-145 cells. After 1 h, the supernatant was replaced with DMEM medium (Gibco, USA) supplemented with 2% fetal bovine serum (FBS) and 1% penicillin-streptomycin. The cells were maintained at 37°C with 5% CO_2_. Cytopathic effects (CPEs) were observed daily. After 3 days, the cultures were collected and subjected to three additional blind passages. Pathogens such as porcine circovirus type 2 (PCV2), classical swine fever virus (CSFV), and pseudorabies virus (PRV) were detected in the cultures using RT-PCR or PCR. To confirm the existence of PRRSV, RT-qPCR was employed. Moreover, the virus was identified by an indirect immunofluorescence assay (IFA) using a polyclonal antibody against the GP5 protein as the primary antibody (Bioss, China) [24]. At the designated time point, viral supernatants were collected and titrated by TCID_50_ to generate a growth curve [25]. Finally, the viral supernatants were stored at −80°C.

### 2.3. Genome Sequencing and Phylogenetic Analysis

The supernatant from the sixth passage of the isolate was utilized for genomic sequencing. The whole-genome sequence was obtained from Tanpu Biotechnology Co., Ltd. (Shanghai, China) using metagenomic sequencing. Contigs were assembled using MegAlign and SeqMan software (DNASTAR lnc., USA) to generate a complete genome. Whole-genome sequences of reference strains and the newly isolated virus were aligned using MEGA software (DNASTAR lnc., USA). Molecular evolutionary analyses and phylogenetic tree construction were performed with MEGA software using the neighbor-joining method. The AA sequences of the ORF5 and NSP2 genes were aligned using MegAlign software. In addition, RFLP typing of the ORF5 gene was performed using Snapgene software (Dotmatics, USA).

### 2.4. Recombination Analysis

As previously reported, recombinant events in the isolated virus were detected using RDP4 software (UCT, South Africa) [1]. Reference strains selected for this study include IA/2014/NADC34 (GenBank accession no. MF326985.1), NADC30 (GenBank accession no. JN654459.1), JXA1 (GenBank accession no. EF112445.1), QYYZ (GenBank accession no. JQ308798.1), and VR2332 (GenBank accession no. EF536003.1). Simplot software (ReduSoft Ltd., Germany) was used to further define the recombination events.

### 2.5. Animal Experiments

Six 4-week-old piglets in good health were procured from Sichuan Meishan Wanjiahao Pig Breeding Co., Ltd. (Sichuan, China). The piglets were confirmed negative for PRRSV, PCV2, PRV, and CSFV. They were randomly divided into two groups (*n* = 3) using a computer-based random number generator. The piglets in the control and challenge groups were housed in separate rooms. Those in the challenge group received a 2 mL intranasal injection of the virus (10^5^TCID_50_/mL), while the control group received 2 mL of DMEM medium administered in the same manner. Rectal temperature and clinical symptoms were recorded daily postinfection. Piglets were weighed at 0, 7, 14, and 21 days postinoculation (dpi), and average daily gain was calculated. Clinical symptom scores were assessed weekly according to established criteria [26]. Blood samples, along with nasal, throat, and anal swabs, were collected at 0, 3, 5, 7, 10, 14, and 21 dpi. Viral load in serum and swab samples was quantified using RT-qPCR. The presence of PRRSV N protein in serum was determined using a commercial IDEXX PRRS 2XR enzyme-linked immunosorbent assay (ELISA) kit (IDEXX, USA), with a S/P value ≥ 0.4 indicating positive antibody presence. Concentrations of cytokines (IL-1*β*, IL-6, IL-8, TNF-*α*, IFN-*α*, and IFN-*γ*) in serum were measured using commercially ELISA kits (Jianglai Biotechnology Co., Ltd., China), with the results expressed in pg/mL. Surviving piglets were humanely euthanized at 21 dpi using intravenous injection of sodium pentobarbital (40 mg/kg) (Sinopharm, China). Viral loads in the heart, liver, spleen, lungs, kidney, tonsil, and lymph nodes were also detected by RT-qPCR. Furthermore, lungs and lymph nodes were fixed in 4% paraformaldehyde for hematoxylin and eosin (H&E) staining and immunohistochemistry (IHC) [24]. Carcasses were stored in designated freezers and disposed of by licensed commercial waste disposal companies following the experiments.

### 2.6. Statistical Analysis

Statistical analysis was performed using two-way analysis of variance (ANOVA), followed by multiple comparisons using GraphPad Prism software (San Diego, USA). A *p* − value  < 0.05 was considered statistically significant. Data are presented as mean ± standard deviation (SD), with each experiment conducted in triplicate.

## 3. Results

### 3.1. Sample Collection

From May to June 2022, a pig farm in Sichuan Province experienced an outbreak characterized by miscarriages in sows and mortality in piglets. Necropsies were randomly performed on three piglets, revealing significant lesions in the lungs and lymph nodes. Blood and tissue samples were collected for pathogen detection, and RT-qPCR confirmed PRRSV infection. Additional testing for PCV2, CSFV, and PRV using PCR or RT-PCR yielded negative results (data not shown).

### 3.2. Virus Isolation and Identification

**Figure 1:**
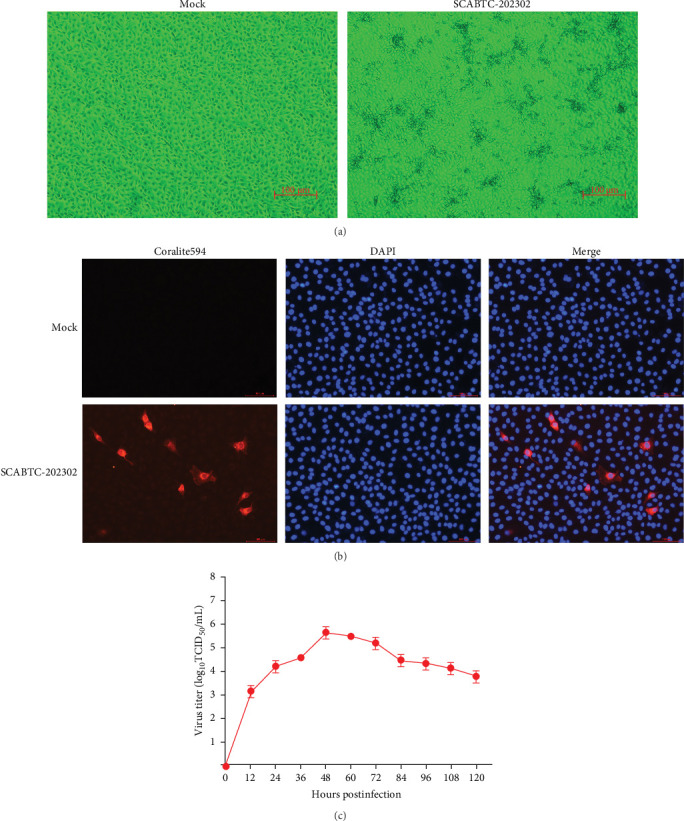
Infection and proliferation of the SCABTC-202302 strain in Marc-145 cells. (A) CPEs in Marc-145 cells were observed at 48 h postinfection (hpi) at 400× magnification, with a scale bar of 100 μm. (B) IFA displayed PRRSV GP5 protein (red fluorescence) in Marc-145 cells at 48 hpi, with nuclei stained by DAPI (blue fluorescence), shown at 400× magnification and a scale bar of 100 μm. (C) Growth curve of the SCABTC-202302 strain in Marc-145 cells. Viral supernatants were collected from 0 to 120 hpi and titrated using the TCID_50_ method. CPEs, cytopathic effects; IFA, immunofluorescence assay; PRRSV, porcine reproductive and respiratory syndrome virus.

Supernatants from PRRSV-positive samples were inoculated into Marc-145 cells, and CPEs were observed during blind passages. Infected cells primarily exhibited rounding and aggregation, with typical cabbage-like lesions appearing at 48 h postinfection (hpi) (Figure 1A). The newly isolated virus, designated SCABTC-202302, was confirmed by RT-qPCR. IFA further validated the virus's presence, showing particular red fluorescence in Marc-145 cells infected with the SCABTC-202302 strain, but not in control cells at 48 hpi (Figure 1B). The multistep growth curve of the virus in Marc-145 cells showed an initial increase in viral titers, peaking at 10^5.66^TCID_50_/mL at 48 hpi, followed by a decline (Figure 1C). These results confirm the successful isolation of the SCABTC-202302 strain.

### 3.3. Genome Sequencing and Phylogenetic Analysis

The complete genome of the SCABTC-202302 strain was determined to be 15,033 bp in length after sequencing and assembly. The genome was annotated based on published PRRSV strains and subsequently uploaded to NCBI, where it was assigned the GenBank accession no. OQ986591.

**Figure 2:**
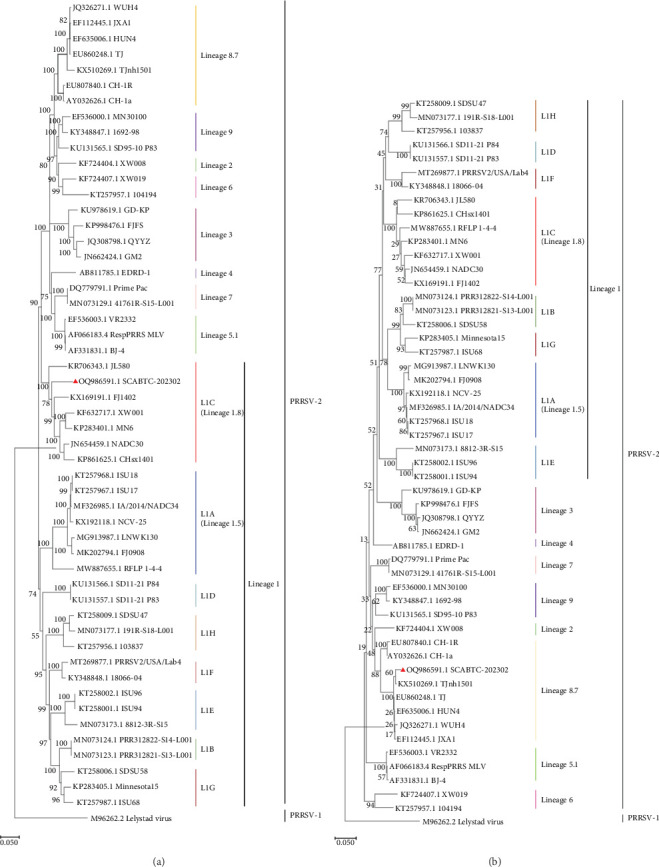
Phylogenetic analysis of the SCABTC-202302 strain. (A) Phylogenetic tree based on the whole genome. (B) Phylogenetic tree based on the ORF5 gene. The lineages of PRRSV-2 and PRRSV-1 are shown, including lineage 1 (sublineages L1A, L1B, L1C, L1D, L1E, L1F, L1G, and L1H), as well as lineages 2, 3, 4, 5.1, 6, 7, 8.7, and 9. The newly isolated strain is marked with a solid red triangle. Phylogenetic trees were constructed using the neighbor-joining method in MEGA software, with 1000 bootstrap replications.

To assess the genetic relationships between the newly isolated SCABTC-202302 strain and PRRSV reference strains downloaded from NCBI (*Supplementary Table 1*), phylogenetic trees were constructed. Whole-genome analysis classified the SCABTC-202302 strain within the L1C lineage (Figure 2A). Phylogenetic analysis of the ORF5 gene identified this strain as belonging to lineage 8.7 (Figure 2B). Multiple sequence alignment of the whole genome showed that the SCABTC-202302 strain shares high nucleotide identity (88.1%−91.5%) with other L1C strains (*Supplementary Table 2*). However, nucleotide alignment of the ORF5 gene indicated 92.4%−97.8% identity with other lineage 8.7 strains (*Supplementary Table 2*). RFLP analysis of the ORF5 gene revealed the following cleavage sites: *Mlu*Ⅰ showed no cleavage site (NA), *Hinc*Ⅱ cleaved at positions 87, 131, and 385 nt, and *Sac*Ⅱ cleaved at positions 49 and 554 nt. Consequently, the RFLP type of the SCABTC-202302 strain is designated as 1-4-3.

**Figure 3:**
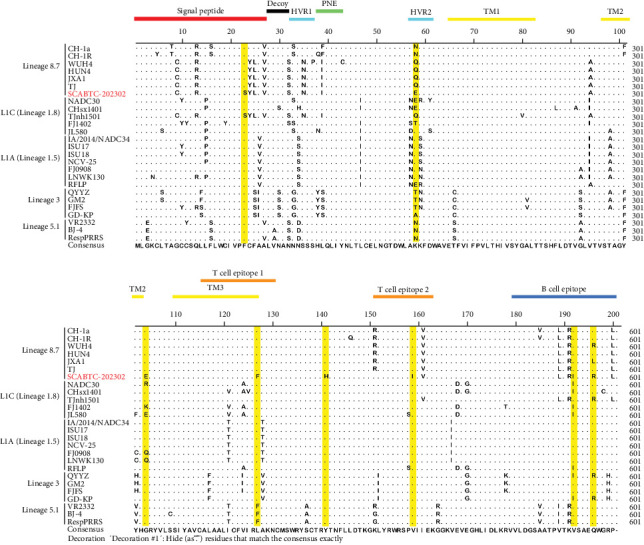
Alignment of ORF5 amino acid sequences. Yellow-highlighted regions indicate mutations specific to the SCABTC-202302 strain.

**Figure 4:**
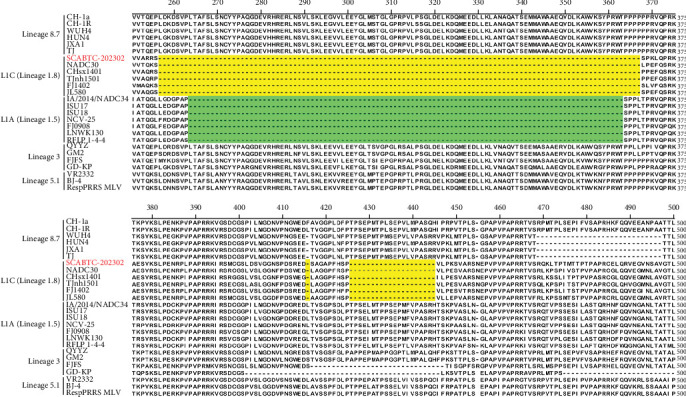
Alignment of NSP2 amino acid sequences. Yellow-highlighted regions indicate deletions specific to L1C, while green-highlighted regions indicate deletions specific to L1A.

To further investigate the genetic diversity of the SCABTC-202302 strain, ORF5 AA sequences were aligned. The results indicated that although most AAs were conserved, several mutations were identified in the signal peptide, hypervariable region (HVR) 2, transmembrane (TM) 2, T cell epitope, and B cell epitope regions. Notably, two distinct AA mutations (Y^141^→H^141^ and V^159^→I^159^) were identified, distinguishing the strain from other representative strains (Figure 3). Alignment of the NSP2 AA sequences revealed a discontinuous 131-AA deletion at positions 257–367, 416, and 426–445, consistent with other L1C strains (Figure 4).

### 3.4. Recombination Analysis

**Figure 5:**
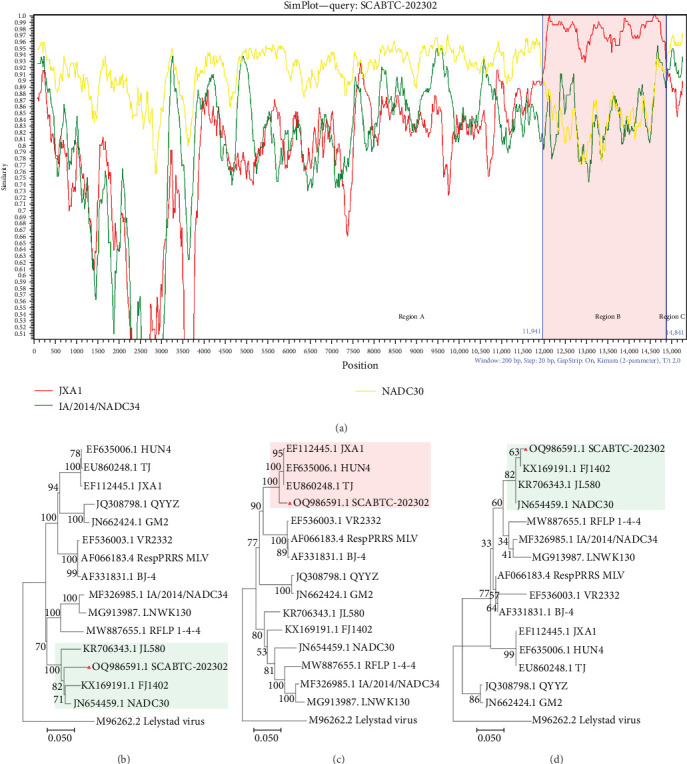
Recombination analysis of the SCABTC-202302 strain. (A) Genome recombination analysis of the SCABTC-202302 strain using SimPlot software. JAX1, NADC30, and IA/2014/NADC34 were used as parental strains. Recombination breakpoints are indicated by blue lines, with their positions labeled in blue at the bottom. The pink region (11941–14841 nt) represents the recombinant segment identified in the SCABTC-202302 strain, while the white regions correspond to parental segments. (B–D) Phylogenetic trees were constructed for each recombinant region (regions A, B, and C) of SCABTC-202302 using the neighbor-joining method in MEGA software, with 1000 bootstrap replications. The SCABTC-202302 strain is marked with a solid red triangle.

The SCABTC-202302 strain was used as the query strain, with VR2332, JXA1, QYYZ, NADC30, and IA/2014/NADC34 serving as reference strains. RDP4 analysis indicated that SCABTC-202302 is a naturally occurring recombinant virus derived from three distinct strains (Table 1 and *Supplementary Figure 2*). This recombination event was further confirmed using SimPlot software (Figure 5A). The major parental strain was NADC30, and the minor parental strain was JXA1. Recombination occurred in the 11,941–14,841 nt region, dividing the genome into three segments. Phylogenetic analyses were conducted in each of these regions. The resulting phylogenetic tree showed that region A of SCABTC-202302 belongs to L1C (Figure 5B), region B to lineage 8.7 (Figure 5C), and region C to L1C (Figure 5D).

### 3.5. Clinical Signs

**Figure 6:**
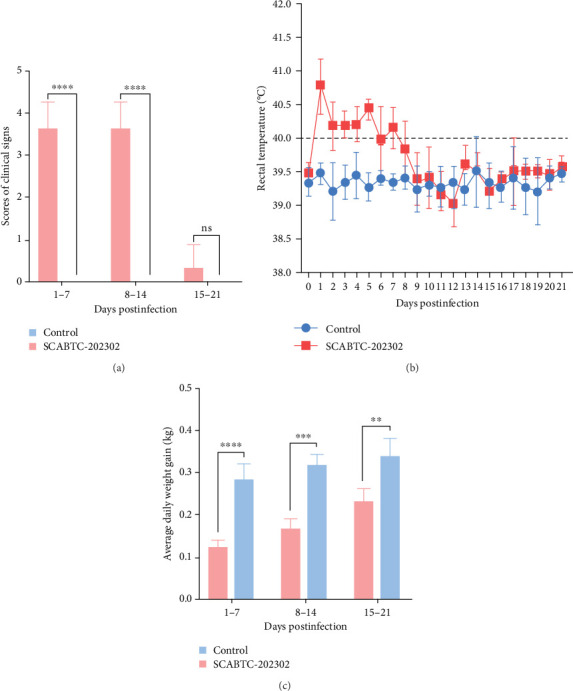
Clinical symptoms in piglets. (A) Clinical scores of piglets. (B) Changes in rectal temperature. (C) Average daily weight gain of piglets. Data are expressed as mean ± SD. Statistical significance is indicated as follows: ns, not significant; *⁣*^*∗*^*p* < 0.05; *⁣*^*∗∗*^*p* < 0.01; *⁣*^*∗∗∗*^*p* < 0.001; *⁣*^*∗∗∗∗*^*p* < 0.0001. SD, standard deviation.

Piglets infected with the SCABTC-202302 strain exhibited persistent fever and anorexia (*Supplementary Figure 3*). A comprehensive statistical analysis of clinical scores revealed that the piglets infected with the SCABTC-202302 strain had significantly higher scores (*p* < 0.0001) compared to the control group during both 1–7 dpi and 8–14 dpi (Figure 6A). Specifically, infected piglets experienced fever (≥40°C) during 1–7 dpi, peaking at 40.7°C on day 1, with temperatures returning to normal by 8 dpi (Figure 6B). Body weight measurements taken at 0, 7, 14, and 21 dpi showed that the average daily gain of SCABTC-202302-infected piglets was significantly lower than that of uninfected piglets (*p* < 0.01) over the infection period (Figure 6C). The control group piglets remained free of clinical signs throughout the experiment, and all piglets survived for the entire duration of the study.

### 3.6. Viremia, Viral Shedding, and Distribution

**Figure 7:**
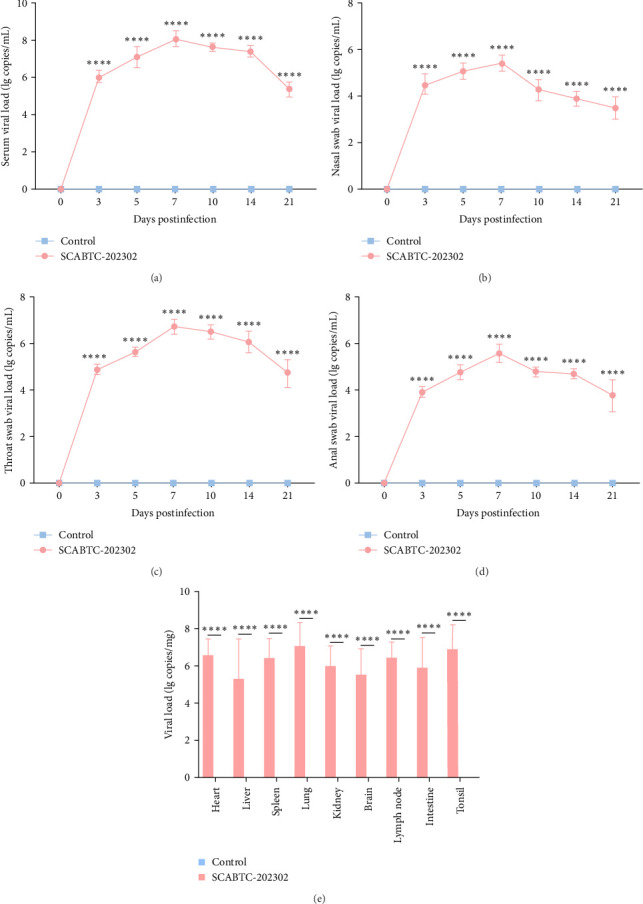
Viremia, viral shedding, and tissue distribution in piglets. (A) Viremia detected in serum. (B) Viral shedding detected in nasal swabs. (C) Viral shedding detected in throat swabs. (D) Viral shedding detected in anal swabs. (E) Viral load measured in various organs. Data are expressed as mean ± SD. Statistical significance is indicated as follows: ns, not significant; *⁣*^*∗*^*p* < 0.05; *⁣*^*∗∗*^*p* < 0.01; *⁣*^*∗∗∗*^*p* < 0.001; *⁣*^*∗∗∗∗*^*p* < 0.0001. SD, standard deviation.

Serum samples were collected at 0, 3, 5, 7, 10, 14, and 21 dpi to measure viral load. In piglets infected with the SCABTC-202302 strain, the serum viral load increased during the first 7 dpi, peaking at 7 dpi (1.3 × 10^8^ copies/mL), before gradually decreasing (Figure 7A). Similarly, viral loads in nasal, throat, and anal swab samples showed a rapid increase followed by a gradual decline. However, the timing of virus shedding and the viral loads detected at different time points varied slightly (Figure 7b–d). Viral loads were also detected in the organs of infected piglets, confirming the presence of the virus in all tissues, with higher concentrations in the lungs, tonsils, and lymph nodes (Figure 7E). No virus was detected in any of the samples from the control group piglets.

### 3.7. Pathological Lesions

**Figure 8:**
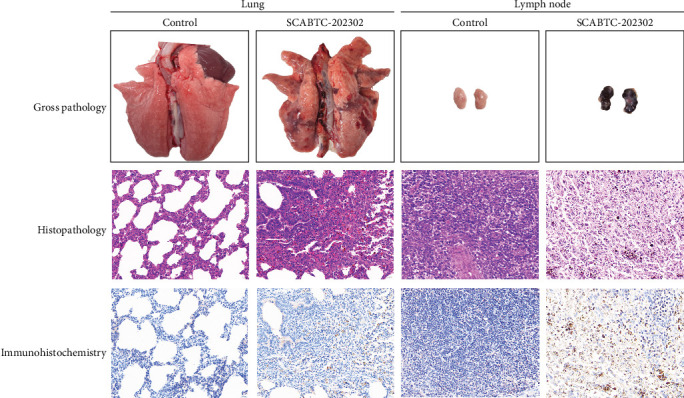
Gross lesions, histopathology, and IHC analysis of the lung and lymph node in piglets at 21 dpi. H&E-stained images were observed at 400× magnification, with a scale bar of 100 μm. In IHC-stained images, brown particles represent the PRRSV GP5 protein, also shown at 400× magnification with a scale bar of 100 μm. H&E, hematoxylin and eosin; IHC, immunohistochemistry.

All surviving piglets were humanely euthanized at 21 dpi for pathological examination. Piglets infected with the SCABTC-202302 strain exhibited extensive lung consolidation, interstitial pneumonia with hemorrhage, and lymph node hemorrhaging (Figure 8). Histopathologic analysis revealed significant inflammatory cell infiltration in the lungs, alveolar epithelial hyperplasia, widened alveolar spaces, and capillary bleeding. In the lymph nodes, hemorrhage, lymphocyte depletion, extensive cell necrosis, and scattered cell debris were observed (Figure 8). Additionally, IHC confirmed the presence of viral antigens in the lungs and lymph nodes of the infected piglets (Figure 8). No pathological lesions or viral antigens were detected in the control group piglets.

### 3.8. Antibody Detection

**Figure 9:**
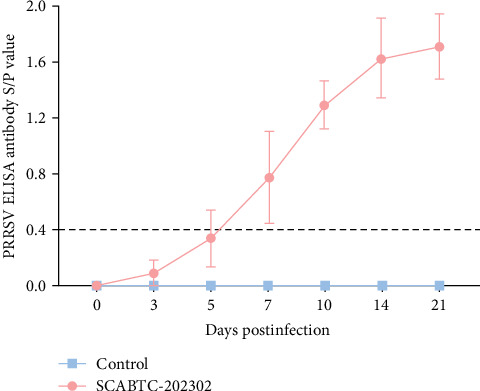
Detection of PRRSV antibodies in piglet serum. PRRSV N protein antibodies were measured using the IDEXX PRRS 2XR ELISA kit. An S/P ratio ≥ 0.4 was considered evidence of positive seroconversion. Data are expressed as mean ± SD. PRRSV, porcine reproductive and respiratory syndrome virus.

Analysis of antibody levels against the PRRSV N protein revealed that the piglets infected with the SCABTC-202302 strain underwent seroconversion (S/P > 0.4) by 7 dpi. As time progressed, antibody levels continued to increase. In contrast, the control piglets consistently showed negative antibody levels (S/P < 0.4) (Figure 9).

### 3.9. Cytokines Determination

**Figure 10:**
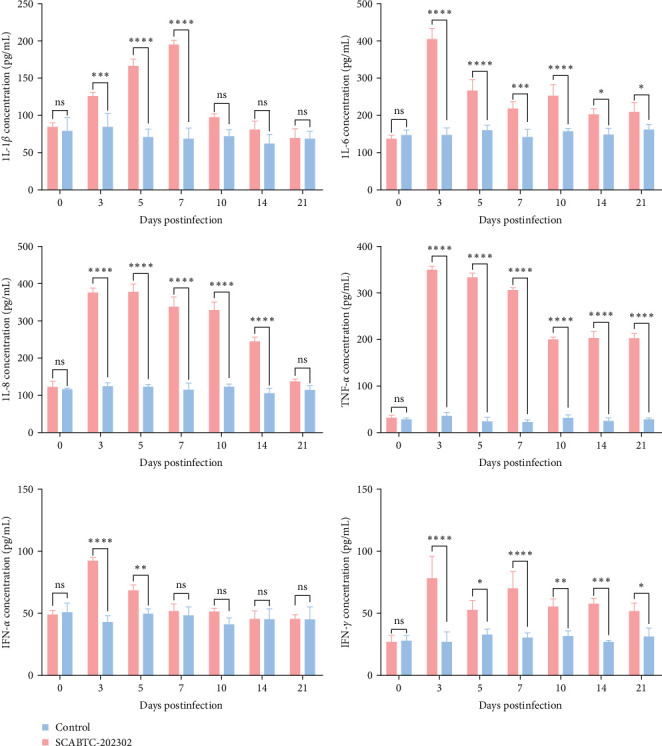
Cytokine levels in the serum of piglets. The levels of IL-1*β*, IL-6, IL-8, TNF-*α*, IFN-*α*, and IFN-*γ* were measured using ELISA. Data are expressed as mean ± SD. Statistical significance is indicated as follows: ns, not significant; *⁣*^*∗*^*p* < 0.05; *⁣*^*∗∗*^*p* < 0.01; *⁣*^*∗∗∗*^*p* < 0.001; *⁣*^*∗∗∗∗*^*p* < 0.0001.

To confirm that SCABTC-202302 infection induces inflammatory responses in piglets, we analyzed serum cytokine levels. The levels of IL-1*β*, IL-6, IL-8, TNF-*α*, IFN-*α*, and IFN-*γ* from 0 to 21 dpi are shown in Figure 10. In piglets infected with the SCABTC-202302 strain, IL-1*β* levels were 85.16 pg/mL at 0 dpi, gradually increasing from 3 dpi and peaking at 7 dpi (196 pg/mL). Levels then sharply declined at 10 dpi, returning near baseline (98.26 pg/mL), and remained stable until 21 dpi. IL−6 levels rose significantly (*p* < 0.0001) at 3 dpi, reaching a peak of 406.72 pg/mL, followed by a gradual decrease. A brief increase occurred at 10 dpi, but levels continued to decline through 21 dpi. Similarly, IL-8 levels increased significantly (*p* < 0.0001) at 3 dpi, reaching 377.11 pg/mL and peaking at 5 dpi (379.24 pg/mL). Levels then gradually decreased, approaching preinfection levels (139.41 pg/mL) at 21 dpi. TNF-*α* levels rose significantly (*p* < 0.0001) at 3 dpi, peaking at 350.98 pg/mL. Levels then decreased gradually, with a notable drop to 201.61 pg/mL at 10 dpi, remaining stable thereafter. IFN-*α* levels increased significantly (*p* < 0.01) from 3 to 5 dpi, peaking at 3 dpi (92.78 pg/mL), then gradually decreased to preinfection levels (52.24 pg/mL) at 7 dpi and remained steady until 21 dpi. IFN-*γ* levels in infected piglets also increased significantly (*p* < 0.0001) at 3 dpi, peaking at 78.42 pg/mL, followed by a decline with a brief increase observed at 7 dpi before further decrease. In general, serum cytokine levels in piglets infected with the SCABTC-202302 strain exhibited distinct trends throughout the infection period. In contrast, control piglets displayed no significant changes in IL-1*β*, IL-6, IL-8, TNF-*α*, IFN-*α*, and IFN-*γ* levels throughout the experiment (Figure 10).

## 4. Discussion

PRRSV is a crucial pathogen in swine disease, causing significant economic losses to the global pig industry [1]. The existence of various lineages complicates the epidemiological landscape of PRRSV [27]. Recently, LlC strains have emerged as the dominant lineage in China [28]. LlC strains are prone to recombination with other PRRSV strains, influencing both genetic diversity and virulence of PRRSV isolates [29]. In the current study, a novel natural recombinant L1C variant, SCABTC-202302, was isolated. Its genetic characteristics, recombination events, pathogenicity, immune response, and antibody response were further investigated.

Phylogenetic analysis of the whole genome and ORF5 gene classified the SCABTC-202302 strain within the LlC and lineage 8.7, respectively. Whole-genome nucleotide sequence analysis revealed a high identity (88.1%−91.5%) between SCABTC-202302 and other L1C strains, while ORF5 gene analysis showed a high identity (92.4%−97.8%) between SCABTC-202302 and other lineage 8.7 strains. Additionally, a 131-AA deletion in the NSP2 gene, characteristic of L1C strains, was identified in SCABTC-202302. These results suggest that ORF5 gene analysis alone may not be sufficient for PRRSV strain classification, and both whole genome and NSP2 gene should be considered as important references. In addition, alignment of the ORF5 AA sequence in SCABTC-202302 revealed notable differences in the signal peptide, HVR2, TM2, TM3, T cell epitope, and B cell epitope regions. Importantly, two previously unreported AA mutations (Y^141^→H^141^ and V^159^→I^159^) were identified, highlighting the complex variability of PRRSV strains and suggesting potential associations with virulence and immune evasion [19]. These results indicate that SCABTC-202302 is a novel L1C variant requiring continued monitoring.

PRRSV frequently undergoes mutations and recombination, significantly contributing to its genomic diversity [27, 30]. The coexistence of different lineages in the field creates favorable conditions for recombination, promoting the emergence of novel epidemic strains [31]. Our recombinant analysis revealed that SCABTC-202302 is a novel recombinant virus, primarily derived from the NADC30 strain, with recombinant elements from the IA/2014/NADC34 and JXA1 strains. Specifically, the ORF2–ORF7 region was derived from JXA1, which explains the discrepancies observed between the phylogenetic analyses of the ORF5 gene and the complete genome in this study. Previous studies have indicated that PRRSV recombination events typically occur in the NSP3–NSP9 and ORF2–ORF6 regions [1, 32]. However, in this study, a recombination event was identified in the ORF2–ORF7 region. Notably, unlike previous reports where the JXA1 strain provided nonstructural protein regions, the recombinant SCABTC-202302 strain incorporates structural protein regions from the JXA1 strain. These recombination events may affect viral replication, virulence, and immune evasion [33–35]. Consequently, the natural recombination of PRRSV strains poses a significant challenge to effective disease prevention and control.

The pathogenicity of recombinant PPRSV strains tends to fall within the range of their parental strains [20, 27]. Clinically, the SCABTC-202302 strain has been associated with a 16% abortion rate in sows and a 7% mortality rate in piglets. Current research indicates that this strain exhibits moderate pathogenicity in piglets, causing persistent fever and significantly reduced weight gain. Moreover, severe lung consolidation and lymph node hemorrhaging were observed, with histopathological analysis revealing pronounced interstitial pneumonia. The virus was detected in all organs, resulting in sustained high-level viremia in the piglets. However, no deaths occurred during the study. Various factors, including genetic recombination, mutation, and environmental influence, contribute to the differences in the pathogenicity of PRRSV strains [29]. Further research is needed to understand the causes of clinical deaths in piglets infected with the SCABTC-202302 strain. Several studies have also assessed the pathogenicity of the L1C strain and variants, which are prevalent in China, finding their pathogenicity to range from moderate to high. Research by Zhao et al. [36] confirmed that the L1C strain JL580 is a recombinant strain of NADC30 and HP-PRRSV, exhibiting high pathogenicity and causing 100% mortality in piglets under experimental infection conditions. Lei et al. [37] reported that the L1C strain CHsx1401 caused a severe PRRS outbreak in an intensive pig farm in Shanxi, leading to abortions and stillbirths in sows and a mortality rate exceeding 30% among piglets. Zhang et al. [32] found that piglets infected with the L1C strain SD53-1603 displayed noticeable clinical symptoms, such as coughing and anorexia, along with typical pathological changes, though with lower pathogenicity compared to HP-PRRSV. Differences in the pathogenic characteristics of L1C strains may explain why current commercial PRRS vaccines do not fully protect against them, allowing the virus to continue spreading in China [38]. Overall, recombination, mutation, and pathogenicity variation in L1C strains have contributed to their persistence in pig populations. Continuous monitoring of L1C strains' epidemiology and developing safe, effective vaccines are essential to control their spread.

PRRSV infection usually triggers inflammatory responses [21, 39]. In this study, elevated levels of IL-6, TNF-*α*, and IFN-*γ* were detected in the serum of piglets infected with the SCABTC-202302 strain from 3 to 21 dpi. IL-6 plays a vital role in managing the acute inflammation, with increased concentrations associated with systemic and respiratory manifestations [22]. TNF-*α*, a prominent pro-inflammatory cytokine, is essential for initiating the innate immune response and coordinating the adaptive immune response [40]. IFN-*γ*, produced by various immune cells, regulates immune and inflammatory responses [41]. The levels of IL-6, TNF-*α*, and IFN-*γ* were significantly correlated with viremia and tissue viral load [21, 22]. Additionally, elevated IL-8 levels were observed from 3 to 21 dpi in the serum of piglets infected with SCABTC-202302. IL-8 is a pro-inflammatory cytokine that attracts neutrophils to inflammation sites [21]. Higher levels of IL-1*β* and IFN-*α* observed from 3 to 5 dpi may be related to the hyperthermia seen postinfection [21]. Excessive production of pro-inflammatory cytokines, including IFN-*γ*, IL-1*β*, and IL-8, in the early stages of infection with virulent PRRSV strains is a major factor contributing to pulmonary damage [39, 42]. These findings indicate a severe inflammatory response in piglets infected with the SCABTC-202302 strain, especially in the early stages of infection, which may partly explain the clinical deaths of piglets.

A major limitation of this study is the lack of a systematic retrospective investigation and tracing of SCABTC-202302, which hinders a comprehensive understanding of the epidemiology and circulation patterns of L1C strains in Sichuan, China. As such, continuous long-term monitoring and epidemiological studies of PRRSV are essential. Additionally, while our study has elucidated the genetic characteristics, pathogenicity, and immunological profile of SCABTC-202302, the analysis was limited to a single strain. Additional studies are needed to further expand the available data on L1C strains.

In summary, we isolated a natural recombinant L1C variant, SCABTC-202302, with an RFLP pattern of 1-4-3. We investigated its genetic evolution, recombination events, pathogenicity, and immune response and antibody response. The strain is a novel recombinant virus, with NADC30 serving as the primary parent and JXA1 contributing recombinant fragments. It caused lung and lymph node damage, prolonged high-level viremia, and elevated inflammatory factors in infected piglets. Our findings contribute to a deeper understanding of the genetic diversity and pathogenic mechanisms of L1C strains, enriching the PRRSV pathogen database. In the future, enhancing PRRSV monitoring and developing broad-spectrum vaccines targeting to multiple lineages will be crucial for effective PRRSV prevention and control.

## Figures and Tables

**Table 1:** Recombination analysis of the SCABTC-202302 strain using RDP4 software.

Recombinant strain	Major parental strain	Minor parental strain	Recombinant breakpoint (nt)	The *p*-value of the recombination analysis method						
				RDP	GENECONV	BootScan	MaxChi	Chimaera	SiScan	3seq
SCABTC−202302	IA/2014/NADC34	JAX1	10,490–11,675	4.576 × 10^−5^	NS	2.837 × 10^−4^	5.919 × 10^−9^	1.230 × 10^−7^	1.021 × 10^−6^	NS
	NADC30	JAX1	11,688–14,603	1.461 × 10^−165^	4.858 × 10^−149^	9.109 × 10^−169^	2.083 × 10^−40^	2.244 × 10^−41^	1.734 × 10^−48^	8.884 × 10^−136^

Abbreviation: NS, no significant *p*-value.

## Data Availability

The data that support the findings of this study are available from the corresponding author upon reasonable request.

## Supporting Information

Supporting Information Table S1: Reference strains used in the phylogenetic analysis for this study. Table S2: Nucleotide sequence identity of the whole genome and ORF5 gene between the SCABTC-202302 strain and the reference strains. Figure S1: Clinical images of pigs naturally infected with the SCABTC-202302 strain. (A) The sow gave birth to mummified fetuses. (B) The piglets are dying. Figure S2: Prediction of recombination events in the SCABTC-202302 strain using RDP4 software. (A) The first recombination breakpoint is located in the 10490-11675 nt region (pink region), with IA/2014/NADC34 and JXA1 serving as the major and minor parents, respectively. (B) The second recombination breakpoint is located in the 11688-14603 nt region (pink region), with NADC30 and JXA1 as the major and minor parents, respectively. Figure S3: External pictures of piglets in this study. (A) Piglets of the control group in the animal experiments. (B) Piglets of the SCABTC-202302-infected group in the animal experiments.

## References

[1] Xu H., Zhang S., Guo Z. (2024). Novel Characterization of NADC30-Like and NADC34-like PRRSV Strains in China: Epidemiological Status and Pathogenicity Analysis of L1A Variants1. *Journal of Integrative Agriculture*.

[2] Xia D.-S., Chang T., Huang X.-Y. (2023). Isolation, Pathogenicity, and Comparative Phylogenetic Characteristics of an Intralineage Recombinant NADC34-Like PRRSV in China. *Transboundary and Emerging Diseases*.

[3] Brinton M. A., Gulyaeva A. A., Balasuriya U. B. (2021). ICTV Virus Taxonomy Profile: Arteriviridae 2021. *Journal of General Virology*.

[4] Ying F., T.Emmely E., Yanhua L. (2012). Efficient—2 Frameshifting By Mammalian Ribosomes to Synthesize an Additional Arterivirus Protein. *Proceedings of the National Academy of Sciences of the United States of America*.

[5] Benfield D. A., Nelson E., Collins J. E. (1992). Characterization of Swine Infertility and Respiratory Syndrome (SIRS) Virus (Isolate ATCC VR-2332). *Journal of Veterinary Diagnostic Investigation*.

[6] Wensvoort G., Terpstra C., Pol J. (1991). Mystery Swine Disease in the Netherlands: The Isolation of Lelystad Virus. *Veterinary Quarterly*.

[7] Sun Q., Xu H., Li C., Li W., Zhang H., Tian Z. (2022). Advances in Researches on Classification and Differences Between PRRSV-1 and PRRSV-2. *Chinese Journal of Preventive Veterinary Medicine*.

[8] Zhang R., Li H., Xie H. (2024). Comparing the Molecular Evolution and Recombination Patterns of Predominant PRRSV-2 Lineages Co-Circulating in China. *Frontiers in Microbiology*.

[9] Luo Q., Zheng Y., He Y. (2023). Genetic Variation and Recombination Analysis of the GP5 (GP5a) Gene of PRRSV-2 Strains in China From 1996 to 2022. *Frontiers in Microbiology*.

[10] Shi M., Lam T. T.-Y., Hon C.-C. (2010). Phylogeny-Based Evolutionary, Demographical, and Geographical Dissection of North American Type 2 Porcine Reproductive and Respiratory Syndrome Viruses. *Journal of Virology*.

[11] Paploski I. A., Pamornchainavakul N., Makau D. N. (2021). Phylogenetic Structure and Sequential Dominance of Sub-Lineages of PRRSV Type-2 Lineage 1 in the United States. *Vaccines*.

[12] Wesley R. D., Mengeling W. L., Lager K. M., Clouser D. F., Landgraf J. G., Frey M. L. (1998). Differentiation of a Porcine Reproductive and Respiratory Syndrome Virus Vaccine Strain From North American Field Strains By Restriction Fragment Length Polymorphism Analysis of ORF 5. *Journal of Veterinary Diagnostic Investigation*.

[13] Guo Z., Chen X.-X., Li X., Qiao S., Deng R., Zhang G. (2019). Prevalence and Genetic Characteristics of Porcine Reproductive and Respiratory Syndrome Virus in Central China During 2016–2017: NADC30-Like PRRSVs Are Predominant. *Microbial Pathogenesis*.

[14] Xu H., Song S., Zhao J. (2020). A Potential Endemic Strain in China: NADC34-Like Porcine Reproductive and Respiratory Syndrome Virus. *Transboundary and Emerging Diseases*.

[15] Tian K., Yu X., Zhao T. (2007). Emergence of Fatal PRRSV Variants: Unparalleled Outbreaks of Atypical PRRS in China and Molecular Dissection of the Unique Hallmark. *PLoS ONE*.

[16] Li C., Zhuang J., Wang J. (2016). Outbreak Investigation of NADC30-Like PRRSV in South-East China. *Transboundary and Emerging Diseases*.

[17] Zhao H. Z., Liu C. Y., Meng H. (2024). Evolution Characterization and Pathogenicity of an NADC34-Like PRRSV Isolated From Inner Mongolia, China. *Viruses*.

[18] Huang B., Deng L., Xu T. (2024). Isolation and Pathogenicity Comparison of Two Novel Natural Recombinant Porcine Reproductive and Respiratory Syndrome Viruses With Different Recombination Patterns in Southwest China. *Microbiology Spectrum*.

[19] Tu T., Li Y., Zhang G. (2024). Isolation, Identification, Recombination Analysis and Pathogenicity Experiment of a PRRSV Recombinant Strain in Sichuan Province, China. *Frontiers in Microbiology*.

[20] Yu Y., Zhang Q., Cao Z. (2022). Recent Advances in Porcine Reproductive and Respiratory Syndrome Virus NADC30-Like Research in China: Molecular Characterization, Pathogenicity, and Control. *Frontiers in Microbiology*.

[21] Renson P., Rose N., Dimna M. Le (2017). Dynamic Changes in Bronchoalveolar Macrophages and Cytokines During Infection of Pigs With a Highly or Low Pathogenic Genotype 1 PRRSV Strain. *Veterinary Research*.

[22] Sánchez-Carvajal J., Rodríguez-Gómez I., Ruedas-Torres I. (2020). Activation of Pro- and Anti-Inflammatory Responses in Lung Tissue Injury During the Acute Phase of PRRSV-1 Infection With the Virulent Strain Lena. *Veterinary Microbiology*.

[23] Lunney J. K., Fang Y., Ladinig A. (2016). Porcine Reproductive and Respiratory Syndrome Virus (PRRSV): Pathogenesis and Interaction With the Immune System. *Annual Review of Animal Biosciences*.

[24] Zhao J., Zhu L., Xu L. (2022). The Construction and Immunogenicity Analyses of Recombinant Pseudorabies Virus With NADC30-Like Porcine Reproductive and Respiratory Syndrome Virus-Like Particles Co-expression. *Frontiers in Microbiology*.

[25] Reed L. J., Muench H. (1938). A Simple Method of Estimation of Fifty Percent Endpoint. *American Journal of Epidemiology*.

[26] Li Y., Zhou L., Zhang J. (2014). Nsp9 and Nsp10 Contribute to the Fatal Virulence of Highly Pathogenic Porcine Reproductive and Respiratory Syndrome Virus Emerging in China. *PLoS Pathogens*.

[27] Han G., Lei K., Xu H., He F. (2020). Genetic Characterization of a Novel Recombinant PRRSV2 From Lineage 8, 1 and 3 in China With Significant Variation in Replication Efficiency and Cytopathic Effects. *Transboundary and Emerging Diseases*.

[28] Tian K. (2017). NADC30-Like Porcine Reproductive and Respiratory Syndrome in China. *The Open Virology Journal*.

[29] Rawal G., Almeida M. N., Gauger P. C. (2023). In Vivo and In Vitro Characterization of the Recently Emergent PRRSV 1-4-4 L1C Variant (L1C.5) in Comparison With Other PRRSV-2 Lineage 1 Isolates. *Viruses*.

[30] Han W., Wu J.-J., Deng X.-Y. (2009). Molecular Mutations Associated With the in Vitro Passage of Virulent Porcine Reproductive and Respiratory Syndrome Virus. *Virus Genes*.

[31] Park J., Choi S., Jeon J. H., Lee K.-W., Lee C. (2020). Novel Lineage 1 Recombinants of Porcine Reproductive and Respiratory Syndrome Virus Isolated From Vaccinated Herds: Genome Sequences and Cytokine Production Profiles. *Archives of Virology*.

[32] Zhang H., Leng C., Ding Y. (2019). Characterization of Newly Emerged NADC30-Like Strains of Porcine Reproductive and Respiratory Syndrome Virus in China. *Archives of Virology*.

[33] Veit M., Matczuk A. K., Sinhadri B. C., Krause E., Thaa B. (2014). Membrane Proteins of Arterivirus Particles: Structure, Topology, Processing and Function. *Virus Research*.

[34] Kimpston-Burkgren K., Correas I., Osorio F. A. (2017). Relative Contribution of Porcine Reproductive and Respiratory Syndrome Virus Open Reading Frames 2–4 to the Induction of Protective Immunity. *Vaccine*.

[35] Fang Y., Snijder E. J. (2010). The PRRSV Replicase: Exploring the Multifunctionality of an Intriguing Set of Nonstructural Proteins. *Virus Research*.

[36] Zhao K., Ye C., Chang X. B. (2015). Importation and Recombination are Responsible for the Latest Emergence of Highly Pathogenic PRRSV in China. *Journal of Virology*.

[37] Lei Z., Zichun W., Yuping D., Xinna G., Xin G., Hanchun Y. (2015). NADC30-Like Strain of Porcine Reproductive and Respiratory Syndrome Virus, China. *Emerging Infectious Diseases*.

[38] Chen X. X., Zhou X., Guo T., Qiao S., Zhang G. (2021). Efficacy of a Live Attenuated Highly Pathogenic PRRSV Vaccine Against a NADC30-Like Strain Challenge: Implications for ADE of PRRSV. *BMC Veterinary Research*.

[39] Liu Y., Shi W., Zhou E. (2010). Dynamic Changes in Inflammatory Cytokines in Pigs Infected With Highly Pathogenic Porcine Reproductive and Respiratory Syndrome Virus. *Clinical and Vaccine Immunology*.

[40] Borghetti P., Saleri R., Ferrari L. (2011). Cytokine Expression, Glucocorticoid and Growth Hormone Changes After Porcine Reproductive and Respiratory Syndrome Virus (PRRSV-1) Infection in Vaccinated and Unvaccinated Naturally Exposed Pigs. *Comparative Immunology, Microbiology and Infectious Diseases*.

[41] Gerner W., Talker S. C., Koinig H. C., Sedlak C., Mair K. H., Saalmüller A. (2015). Phenotypic and Functional Differentiation of Porcine *αβ* T Cells: Current Knowledge and Available Tools. *Molecular Immunology*.

[42] Weesendorp E., Morgan S., Stockhofe-Zurwieden N., Graaf D. J. Popma-De, Graham S. P., Rebel J. M. (2013). Comparative Analysis of Immune Responses Following Experimental Infection of Pigs with European Porcine Reproductive and Respiratory Syndrome Virus Strains of Differing Virulence. *Veterinary Microbiology*.

