# Supplementary material for: A RFLP 1-4-3 L1C Variant of PRRSV-2 Isolated in Sichuan Province, China: Genetic Characterization and Pathogenicity
Source: Transbound Emerg Dis. 2024 Dec 12;2024:6817783. doi: 10.1155/tbed/6817783 (PMC12017144; doi:10.1155/tbed/6817783)
Supplement: Supporting Information — Table S1: Reference strains used in the phylogenetic analysis for this study. Table S2: Nucleotide sequence identity of the whole genome and ORF5 gene between the SCABTC-202302 strain and the reference strains. Figure S1: Clinical images of pigs naturally infected with the SCABTC-202302 strain. (A) The sow gave birth to mummified fetuses. (B) The piglets are dying. Figure S2: Prediction of recombination events in the SCABTC-202302 strain using RDP4 software. (A) The first recombination breakpoint is located in the 10490-11675 nt region (pink region), with IA/2014/NADC34 and JXA1 serving as the major and minor parents, respectively. (B) The second recombination breakpoint is located in the 11688-14603 nt region (pink region), with NADC30 and JXA1 as the major and minor parents, respectively. Figure S3: External pictures of piglets in this study. (A) Piglets of the control group in the animal experiments. (B) Piglets of the SCABTC-202302-infected group in the animal experiments. [file 6817783.f1.docx]

**Supplementary Tables**

**Supplementary Table 1** Reference strains used in the phylogenetic analysis for this study

| **Number** | **Strain name** | **Country** | **Isolation date** | **GenBank accession number** |
| --- | --- | --- | --- | --- |
| 1 | JL580 | China | 2013 | KR706343.1 |
| 2 | FJ1402 | China | 2014 | KX169191.1 |
| 3 | XW001 | USA | 2012 | KF632717.1 |
| 4 | MN6 | USA | 2012 | KP283401.1 |
| 5 | NADC30 | USA | 2008 | JN654459.1 |
| 6 | CHsx1401 | China | 2014 | KP861625.1 |
| 7 | ISU18 | USA | 2014 | KT257968.1 |
| 8 | ISU17 | USA | 2014 | KT257967.1 |
| 9 | IA/2014/NADC34 | USA | 2014 | MF326985.1 |
| 10 | NCV-25 | USA | 2016 | KX192118.1 |
| 11 | LNWK130 | China | 2017 | MG913987.1 |
| 12 | FJ0908 | China | 2018 | MK202794.1 |
| 13 | RFLP 1-4-4 | USA | 2020 | MW887655.1 |
| 14 | SD11-21_P84 | USA | 2011 | KU131566.1 |
| 15 | SD11-21 P83 | USA | 2011 | KU131557.1 |
| 16 | SDSU47 | USA | 2014 | KT258009.1 |
| 17 | 191R-S18-L001 | USA | 2017 | MN073177.1 |
| 18 | 103837 | USA | 2014 | KT257956.1 |
| 19 | PRRSV2/USA/Lab4 | USA | 2006 | MT269877.1 |
| 20 | 18066-04 | USA | 2004 | KY348848.1 |
| 21 | ISU96 | USA | 2014 | KT258002.1 |
| 22 | ISU94 | USA | 2014 | KT258001.1 |
| 23 | 8812-3R-S15 | USA | 2018 | MN073173.1 |
| 24 | PRR312822-S14-L001 | USA | 2017 | MN073124.1 |
| 25 | PRR312821-S13-L001 | USA | 2017 | MN073123.1 |
| 26 | SDSU58 | USA | 2014 | KT258006.1 |
| 27 | Minnesota15 | USA | 2012 | KP283405.1 |
| 28 | ISU68 | USA | 2014 | KT257987.1 |
| 29 | XW008 | USA | 2012 | KF724404.1 |
| 30 | GD-KP | China | 2015 | KU978619.1 |
| 31 | FJFS | China | 2015 | KP998476.1 |
| 32 | QYYZ | China | 2011 | JQ308798.1 |
| 33 | GM2 | China | 2011 | JN662424.1 |
| 34 | DRD-1 | Japan | 2013 | AB811785.1 |
| 35 | VR2332 | USA | 2007 | EF536003.1 |
| 36 | RespPRRS MLV | USA | 2005 | AF066183.4 |
| 37 | BJ-4 | China | 2000 | AF331831.1 |
| 38 | XW019 | USA | 2013 | KF724407.1 |
| 39 | 104194 | USA | 2014 | KT257957.1 |
| 40 | Prime Pac | USA | 2006 | DQ779791.1 |
| 41 | 41761R-S15-L001 | USA | 2018 | MN073129.1 |
| 42 | WUH4 | China | 2011 | JQ326271.1 |
| 43 | JXA1 | China | 2006 | EF112445.1 |
| 44 | HUN4 | China | 2007 | EF635006.1 |
| 45 | TJ | China | 2006 | EU860248.1 |
| 46 | TJnh1501 | China | 2016 | KX510269.1 |
| 47 | CH-1R | China | 2008 | EU807840.1 |
| 48 | CH-1a | China | 1998 | AY032626.1 |
| 49 | MN30100 | USA | 2007 | EF536000.1 |
| 50 | 1692-98 | USA | 1998 | KY348847.1 |
| 51 | SD95-10 P83 | USA | 1995 | KU131565.1 |
| 52 | Lelystad virus | Netherlands | 1993 | M96262.2 |

**Supplementary table 2** Nucleotide sequence identity of the whole genome and ORF5 gene between the SCABTC-202302 strain and the reference strains

| **Strain name** | **Lineage** | **GenBank accession number** | **Complete genome identity (%)** | **ORF5 gene identity (%)** |
| --- | --- | --- | --- | --- |
| JL580 | L1C | KR706343.1 | 88.9 | 84.9 |
| FJ1402 | L1C | KX169191.1 | 90.3 | 85.2 |
| XW001 | L1C | KF632717.1 | 88.1 | 85.1 |
| MN6 | L1C | KP283401.1 | 88.6 | 85.4 |
| NADC30 | L1C | JN654459.1 | 91.5 | 85.4 |
| CHsx1401 | L1C | KP861625.1 | 89.9 | 84.6 |
| ISU18 | L1A | KT257968.1 | 83.8 | 85.2 |
| ISU17 | L1A | KT257967.1 | 83.9 | 85.2 |
| IA/2014/NADC34 | L1A | MF326985.1 | 84.0 | 85.6 |
| NCV-25 | L1A | KX192118.1 | 83.7 | 85.1 |
| LNWK130 | L1A | MG913987.1 | 83.5 | 84.9 |
| FJ0908 | L1A | MK202794.1 | 83.6 | 85.2 |
| RFLP 1-4-4 | L1A | MW887655.1 | 84.0 | 85.9 |
| SD11-21_P84 | L1D | KU131566.1 | 83.0 | 85.6 |
| SD11-21 P83 | L1D | KU131557.1 | 83.0 | 85.6 |
| SDSU47 | L1H | KT258009.1 | 84.8 | 85.2 |
| 191R-S18-L001 | L1H | MN073177.1 | 84.6 | 84.7 |
| 103837 | L1H | KT257956.1 | 84.9 | 85.2 |
| PRRSV2/USA/Lab4 | L1F | MT269877.1 | 85.4 | 84.7 |
| 18066-04 | L1F | KY348848.1 | 86.3 | 86.2 |
| ISU96 | L1E | KT258002.1 | 84.5 | 84.6 |
| ISU94 | L1E | KT258001.1 | 84.4 | 84.6 |
| 8812-3R-S15 | L1E | MN073173.1 | 83.4 | 84.2 |
| PRR312822-S14-L001 | L1B | MN073124.1 | 85.6 | 85.7 |
| PRR312821-S13-L001 | L1B | MN073123.1 | 85.6 | 85.7 |
| SDSU58 | L1G | KT258006.1 | 84.8 | 84.1 |
| Minnesota15 | L1G | KP283405.1 | 85.2 | 84.1 |
| ISU68 | L1G | KT257987.1 | 85 | 83.6 |
| XW008 | Lineage 2 | KF724404.1 | 80.6 | 89.2 |
| GD-KP | Lineage 3 | KU978619.1 | 79.9 | 85.1 |
| FJFS | Lineage 3 | KP998476.1 | 79.7 | 81.9 |
| QYYZ | Lineage 3 | JQ308798.1 | 79.6 | 82.3 |
| GM2 | Lineage 3 | JN662424.1 | 79.9 | 81.6 |
| EDRD-1 | Lineage 4 | AB811785.1 | 81.0 | 88.7 |
| VR2332 | Lineage 5.1 | EF536003.1 | 81.6 | 87.1 |
| RespPRRS MLV | Lineage 5.1 | AF066183.4 | 83.0 | 87.1 |
| BJ-4 | Lineage 5.1 | AF331831.1 | 82.5 | 86.6 |
| XW019 | Lineage 6 | KF724407.1 | 81.4 | 84.9 |
| 104194 | Lineage 6 | KT257957.1 | 77.9 | 86.9 |
| Prime Pac | Lineage 7 | DQ779791.1 | 82.3 | 88.4 |
| 41761R-S15-L001 | Lineage 7 | MN073129.1 | 82.3 | 88.7 |
| WUH4 | Lineage 8.7 | JQ326271.1 | 82.7 | 97.0 |
| JXA1 | Lineage 8.7 | EF112445.1 | 82.7 | 97.5 |
| HUN4 | Lineage 8.7 | EF635006.1 | 82.7 | 97.5 |
| TJ | Lineage 8.7 | EU860248.1 | 82.6 | 97.3 |
| TJnh1501 | Lineage 8.7 | KX510269.1 | 84.1 | 97.8 |
| CH-1R | Lineage 8.7 | EU807840.1 | 83.1 | 92.4 |
| CH-1a | Lineage 8.7 | AY032626.1 | 83.2 | 93.2 |
| MN30100 | Lineage 9 | EF536000.1 | 81.3 | 89.6 |
| 1692-98 | Lineage 9 | KY348847.1 | 83.1 | 90.0 |
| SD95-10 P83 | Lineage 9 | KU131565.1 | 82.5 | 89.2 |
| Lelystad virus | PRRSV-1 | M96262.2 | 59.0 | 62.5 |

**Supplementary Figures**


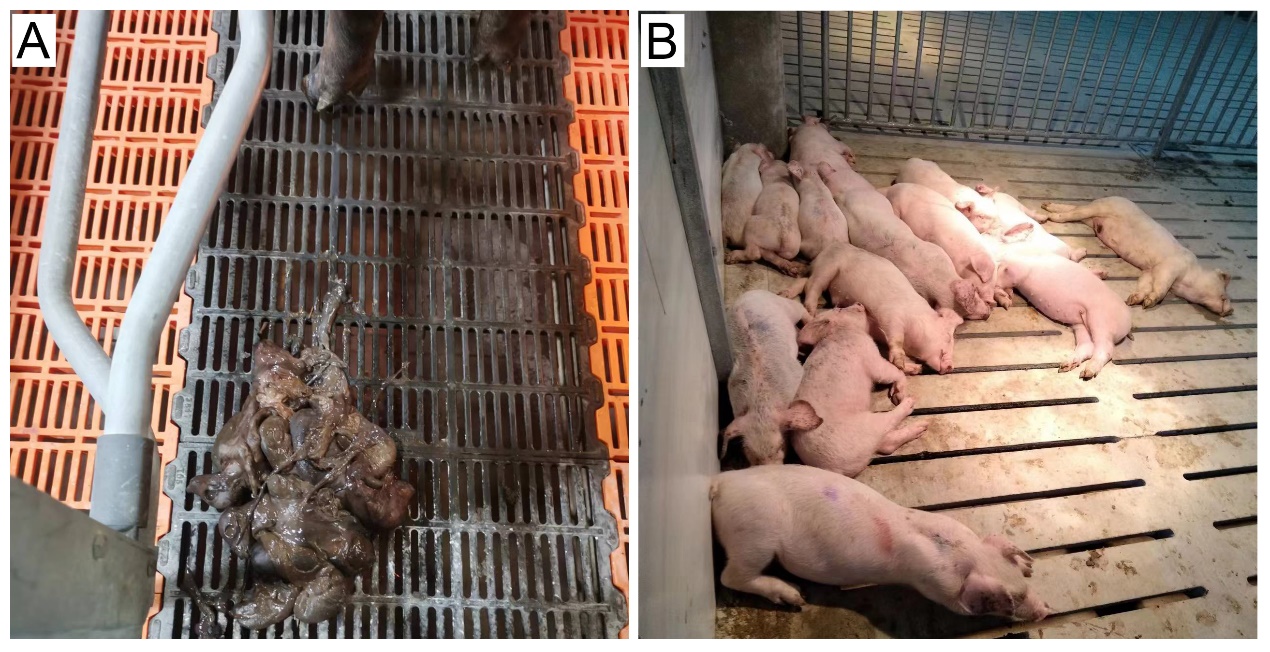


**Supplementary Figure 1** Clinical images of pigs naturally infected with the SCABTC-202302 strain. (A) The sow gave birth to mummified fetuses. (B) The piglets are dying.


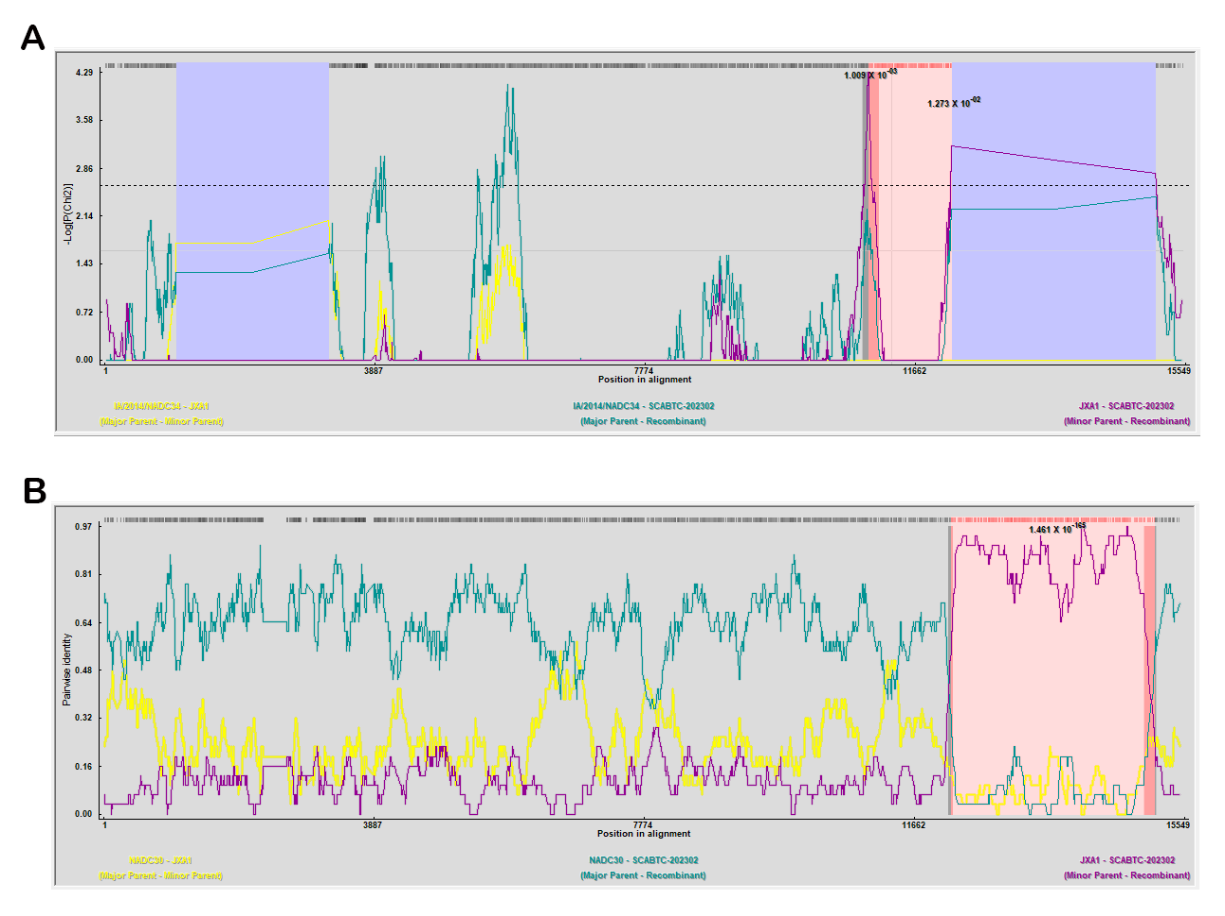


**Supplementary Figure 2** Prediction of recombination events in the SCABTC-202302 strain using RDP4 software. (A) The first recombination breakpoint is located in the 10490-11675 nt region (pink region), with IA/2014/NADC34 and JXA1 serving as the major and minor parents, respectively. (B) Second recombination breakpoint is located in the 11688-14603 nt region (pink region), with NADC30 and JXA1 as the major and minor parents, respectively.


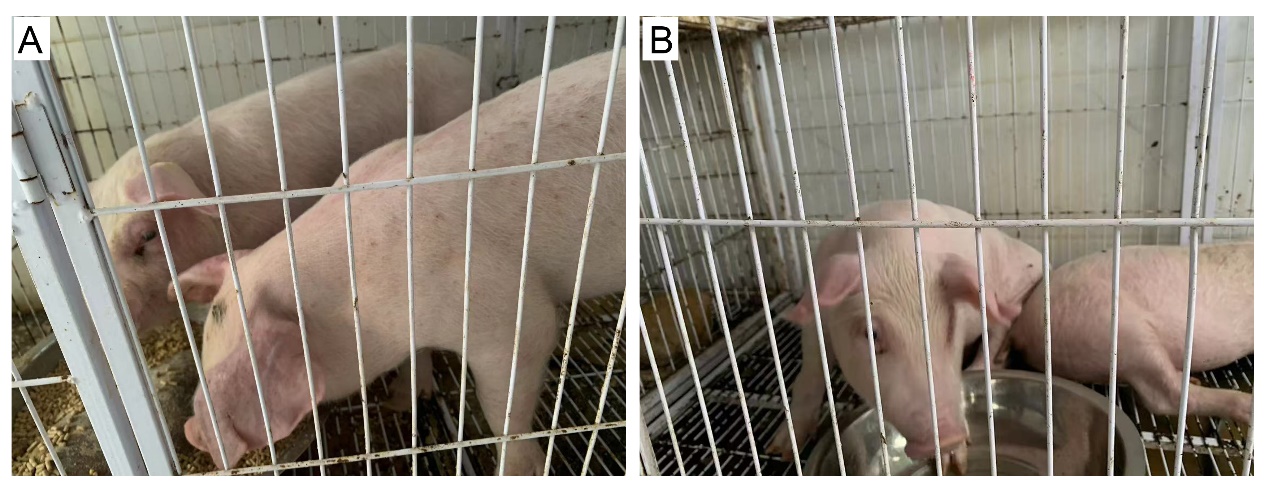


**Supplementary Figure 3** External pictures of pigs from the control group (A) and the SCABTC-202302-infected group (B) in the animal experiments.
